# AIDS-related progressive multifocal leukoencephalopathy in a patient presenting with cerebellar ataxia

**DOI:** 10.1590/0037-8682-0211-2021

**Published:** 2021-06-02

**Authors:** Chee Yik Chang

**Affiliations:** 1 Hospital Sultanah Aminah, Department of General Medicine, Johor, Malaysia.

**FIGURE 1:**
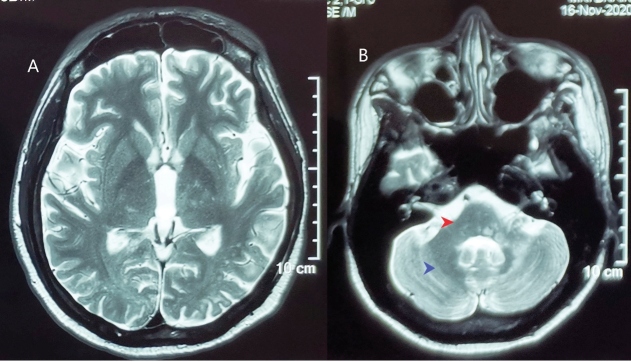
Brain magnetic resonance imaging shows multiple hypointense lesions in **(A)** the bilateral frontal, parietal, and occipital lobes and **(B)** the right cerebellar peduncle (red arrow) and cerebellar hemisphere (blue arrow).

A 30-year-old man with a history of acquired immunodeficiency syndrome (AIDS) (CD4=13 cells/mm^3^) on antiretroviral therapy for one year presented with truncal weakness for four months, followed by involuntary movement of the right upper and lower limbs and slurred speech a month later. He has since started using a wheelchair. Upon examination, he denied symptoms of increased intracranial pressure or prior head trauma. Further examination revealed nystagmus; scanning speech; and intentional tremor, hypertonia, and hyperreflexia of the right upper and lower limbs. Brain magnetic resonance imaging revealed multiple hypointense lesions in the subcortical and deep white matter involving the bilateral parietal, frontal, and occipital lobes; pons; cerebellar peduncle; and cerebellum (Figure 1). Imaging findings suggested progressive multifocal leukoencephalopathy (PML). Lumbar puncture was performed, and JC virus DNA was detected in the cerebrospinal fluid by polymerase chain reaction. Antiretroviral therapy was continued, and the patient was discharged to home after symptom improvement. 

Widespread lesions due to oligodendrocyte infection by the JC virus characterize PML, which is rare and usually associated with AIDS, hematologic malignancies, or immunosuppressive treatments. The availability of antiretroviral therapy has significantly reduced the incidence of PML and its associated mortality. PML typically presents insidiously with focal neurological deficits that vary depending on the location of the lesions^1^. Weakness, speech disturbances, cognitive impairment, headache, gait abnormality, seizures, sensory loss, and visual impairments are among the reported symptoms. PML occurs most commonly in the periventricular and subcortical white matter in the parieto-occipital or frontal lobes. Although rare, lesions in the brainstem, cerebellum, and spinal cord have previously been described^2^. The main treatment for PML in patients with HIV infection is immune restoration with antiretroviral drugs^1^ ^,^ ^2^. 
